# SbLOX3 modulates jasmonate-associated flavonoid accumulation, root growth, and drought-induced root hollow formation in *Scutellaria baicalensis*

**DOI:** 10.3389/fpls.2026.1951407

**Published:** 2026-09-22

**Authors:** Dali Geng, Ya Zhang, Saqi Zhao, Xiaohan Tang, Yuqing Ma, Xiaojun Ma, Xinhong Wang, Jing Shu

**Affiliations:** 1 Department of Forestry Engineering, Shandong Agriculture and Engineering University, Jinan, China; 2 Tea Research Institute, Shandong Academy of Agricultural Sciences, Jinan, China

**Keywords:** 4′-deoxyflavones, drought tolerance, jasmonate, root hollow, SbLOX3

## Abstract

The hollowed root formation and 4′-deoxyflavone accumulation in Scutellaria baicalensis are associated with jasmonate (JAs) signaling, but the upstream regulators integrating these processes with drought tolerance remain unknown. This study investigated whether the 13-lipoxygenase gene SbLOX3 may act as a modulator. Transgenic plants overexpressing SbLOX3 or with RNAi suppression were exposed to polyethylene glycol-simulated and long-term soil drought stress. Metabolite profiling, gene expression analysis, root morphological measurements, and histological observations were conducted. Overexpression of SbLOX3 elevated JAs and 4′-deoxyflavone levels, enhanced root growth and drought tolerance, and induced precocious hollow development in roots under water deficit. In contrast, RNAi lines showed reduced JAs and flavonoid contents, impaired drought tolerance, and absence of hollow formation. SbLOX3 was strongly expressed in xylem, cambium, and phloem under drought. The results suggest that SbLOX3 is associated with a coordinated responseregulate to drought by promoting defensive flavonoid biosynthesis and morphological adaptation, thereby potentially contributing to the accumulation of measured flavonoids.

## 1 Introduction

A member of the Lamiaceae family, *Scutellaria baicalensis* Georgi is a foundational herb in traditional Chinese medicine. Its dried root, known as “Huang-Qin, “ is applied in the treatment of diverse inflammatory and infectious illnesses, a use supported by its potent bioactive compounds (Duan et al., 2014; Liu et al., 2021; Song et al., 2021). The plant’s principal therapeutic effects are ascribed to an array of root-specific 4′-deoxyflavones, chiefly baicalein and wogonin together with their glycosides baicalin and wogonoside (Himeji et al., 2007; Nayak et al., 2014; Zhao et al., 2016; Wang et al., 2018). These specialized flavonoids exhibit a broad spectrum of pharmacological properties—including anticancer, anti-inflammatory, and antiviral activities—making their abundance a key measure of medicinal quality (Himeji et al., 2007; Wang et al., 2018).A distinctive anatomical anomaly known as the “hollowed root” develops in mature *S. baicalensis* plants, particularly those aged more than 2 years. This structure is characterized by the death of secondary xylem cells in the root center, which are eventually surrounded by a layer of protective tertiary phellem tissue (Geng et al., 2023a). The accumulation of baicalein, wogonin, and other 4′-deoxyflavones is significantly higher in roots with a hollow than in those without it (Ren et al., 2009; Zhao et al., 2018). This strong correlation suggests a functional link between the hollowing process and metabolic fortification. The content of methyl jasmonate (MeJA) increases synchronously with the development of root hollow and the expression of flavonoid biosynthetic genes (Geng et al., 2023a). Furthermore, the expression of pathogen- and programmed-cell-death-related genes is induced during this process, suggesting senescence and pathogen defense as two major drivers of hollowed root formation (Geng et al., 2023a).

Jasmonate (JAs) and its derivatives function as core signaling nodes that regulate plant stress responses and secondary metabolism (Wasternack and Strnad, 2018). Application of exogenous methyl jasmonate (MeJA) strongly stimulates the accumulation of baicalein and other 4′-deoxyflavones in *Scutellaria baicalensis*, and endogenous MeJA concentrations rise in parallel with root development and flavonoid gene expression, suggesting that JAs signaling links developmental programming to metabolic fortification (Geng et al., 2023a). This signaling system is highly conserved across land plants. In *Arabidopsis thaliana*, the core JAs perception module—comprising the COI1 receptor, JAsZ repressor proteins, and MYC transcription factors—coordinates responses to wounding, herbivory, and pathogen attack (Yang et al., 2019). JAs also plays essential roles in abiotic stress adaptation. For instance, in Arabidopsis, JAs enhances freezing tolerance through activation of the CBF/DREB1 pathway (Hu et al., 2013; Wang et al., 2021), whereas in rice (*Oryza sativa*), JAs signaling components such as OsbHLH148 and OsJAsZ1 contribute to drought and salinity tolerance (Wang et al., 2021). Moreover, JAs engages in cross-talk with other phytohormones, including abscisic acid (ABA) and gibberellin (GA), to finely adjust growth–defense trade-offs under stress conditions (Yang et al., 2019; Wu et al., 2024).The biosynthesis of JAs is initiated by 13-lipoxygenase (13-LOX), which catalyzes the oxygenation of α-linolenic acid (Wasternack and Strnad, 2018). In *Arabidopsis*, different 13-LOX isoforms have specialized functions: AtLOX3 is involved in wound- and stress-induced JAs production, whereas AtLOX6 contributes to basal JAs levels and responses to salinity stress (Geng et al., 2023b). Recently, *SbLOX3*, encoding a 13-LOX, was identified in *S. baicalensis* roots. Functional studies in hairy root cultures have suggest that *SbLOX3* is instrumental in JAs biosynthesis and its suppression leads to reduced levels of JAs, baicalein, and wogonin (Geng et al., 2023b). Moreover, *SbLOX3* expression is induced by drought stress and pathogen attack, positioning it as a potential upstream sensor linking environmental cues to JAs-mediated defense responses, including flavonoid production (Geng et al., 2023b).

Although the role of *SbLOX3* in JAs and flavonoid biosynthesis has been established in simplified hairy root systems, its function in whole transgenic plants, particularly its role in conferring drought tolerance and its potential interplay with root developmental processes such as hollow formation, remains unexplored. Building upon the foundational knowledge that JAs regulates both hollowed root development and flavonoid accumulation (Geng et al., 2023a) and that *SbLOX3* is a key enzyme for JAs production (Geng et al., 2023b), this study aimed to investigate whether and how *SbLOX3* acts as aregulator in whole *S. baicalensis* plants. We hypothesize that *SbLOX3*-derived JAs signaling is associated withregulateresponse to drought, simultaneously enhancing root hollow development, potentially contributing to the measured flavonoid levels.

## 2 Materials and methods

### 2.1 Plant materials

Tissue-cultured *S. baicalensis* plants were transferred to fresh medium every four weeks and maintained on Murashige and Skoog (MS) medium at 25 °C under a long-day photoperiod (14 h light/10 h dark).

The experiment was conducted in Zibo, Shandong Province. *Scutellaria baicalensis* Georgi was cultivated in pots (20 cm diameter × 60 cm depth) containing a mixture of 90% nursery substrate (0–6 mm; Pindstrup, Denmark) and 10% vermiculite. These containers were buried in the soil inside a greenhouse. Three-month-old tissue-cultured seedlings were used for planting.

Three-month-old tissue-cultured seedlings were transferred to a hydroponic system, where they were placed in plastic containers holding 7 L of Hoagland solution and grown for an additional month before further treatment. Throughout the experiment, plants were maintained in a growth chamber at 25 °C, under a light intensity of 60 μmol m^−2^ s^−1^ and relative humidity of 50%–75%.

### 2.2 Generation of transgenic plants

Transgenic plants were generated by cloning the coding sequences of *SbLOX3* into pDONR207 for sequencing and then into pK7WIWG2D for RNAi and pEarleyGate101 for overexpression based on Gateway recombination technology (Invitrogen, USA).

RNAi and overexpression vectors were constructed following the protocol of Xie et al. (2018) with modification. Transgenic lines of Scutellaria baicalensis were generated using a modified version of the method reported by Zhao et al. (2016). Tissue-cultured leaves served as explants. After wounding the leaf surfaces with a sterile scalpel in an *Agrobacterium tumefaciens* suspension, the leaves were dried on sterile filter paper and placed on MS solid medium containing 50 μM acetosyringone. Incubation proceeded at 25 °C in darkness for 72 h. The explants were then transferred to B5 medium supplemented with 50 mg L^−1^ kanamycin and subcultured every two weeks until transgenic buds emerged. The resulting shoots were confirmed by qRT-PCR.

### 2.3 qRT-PCR analysis

The qRT-PCR analysis was performed using the method described by Geng et al. (2023b). All primers used in this study are listed in **Supplementary Table S1**.

### 2.4 UPLC-MS analysis

All reference standards—baicalin, baicalein, wogonin, wogonoside, pinocembrin, pinocembrin chalcone, chrysin, cinnamic acid methyl jasmonate (MeJA)—were sourced from Desite Biotech (Chengdu, Sichuan, China). JA-Ile were sourced from Solarbio life science (Beijing, China).Stock solutions were prepared at 0.1 mg mL^−1^, and working standard solutions were obtained through serial dilution with methanol. UPLC-MS analysis was carried out following the procedure described by Geng et al. (2023a). UPLC-MS analysis of JA-Ile was carried out following the procedure described below:

JA-Ile was quantified using a Waters ACQUITY UPLC system coupled to an Xevo TQ-S triple-quadrupole mass spectrometer. Separation was performed on a BEH C18 column (2.1 × 100 mm, 1.7 μm) at 40 °C. The mobile phase consisted of 0.1% formic acid in water (A) and 0.1% formic acid in acetonitrile (B) at a flow rate of 0.3 mL min^−1^, with the gradient program: 0–1 min, 5% B; 1–3 min, 5% → 30% B; 3–6 min, 30% → 70% B; 6–8 min, 70% → 95% B; 8–10 min, 95% B; 10–10.5 min, 95% → 5% B; 10.5–12 min, 5% B. The injection volume was 5 μL. The electrospray ionization source was operated in negative mode. Capillary voltage was 2.8 kV, cone voltage was 35 V, source temperature was 120 °C, and desolvation temperature was 350 °C. Quantification was performed in MRM mode using the transitions m/z 322.1 → 130.0 (quantifier) and m/z 322.1 → 154.0 (qualifier). Collision energy was optimized at 20 eV and 18 eV, respectively. The retention time of JA-Ile was approximately 5.8 min under these conditions.

### 2.5 PEG-simulated drought treatment

Hydroponically grown wild-type (WT), RNAi, and overexpressing (OE) seedlings were exposed to drought stress simulated by PEG-8000. The plants were randomly assigned to a control group (without PEG-8000) and a drought group (with 15% PEG-8000), and the stress lasted for 7 days. Root samples were collected at two time points: 6 hours after treatment initiation for qRT-PCR analysis, and 7 days after treatment for quantifying 4′-deoxyflavone levels. All harvested roots were immediately frozen in liquid nitrogen and stored at −80 °C until further analysis.

### 2.6 Long-term drought treatment

36 seedings of WT, RNAi, and OE aged 4 months for root morphology anaylsis and *in situ* hybridization analysis, and 36 seedingling of WT, RNAi, and OE aged 20 months old for root hollow development analysis were assigned to a well-watered control (n = 18) and a long-term drought group (n = 18). Control plants were irrigated to maintain the soil at 75%–85% field capacity, whereas drought-stressed plants were kept at 45%–55% field capacity. The treatment continued for 2 months, after which roots were harvested for subsequent analysis.

### 2.7 Analysis of root morphology

The root morphology was analyized using meathods described by Geng et al. (2018). Ninebiological replicates wereperformed for each measurement.

### 2.8 Root hollow development analysis

After long-term drought stress, seedlings of 20 months old plants in control and drought groups were carefully removed from the soil and washed. Roots were cut at the root-shoot junction, then 3–4-cm segments were excised from the proximal region (immediately below the junction). The root segments were vacuumed packed in FAA stationary solution (5% [v/v] formalin, 5% [v/v] acetic acid, and 90% [v/v] ethyl alcohol) for 30 min and then fixed for 7 days. Fixed root segment were stored at 4°C for paraffin section. Root hollowing was identified by sections based on rules described by Geng et al. (2023a).

### 2.9 Paraffin sectioning and *in situ* hybridization

The Paraffin sectioning and *in situ* hybridization were performed using meathods described by Geng et al. (2018). For root hollow development analysis, all 18 seedings were sectioned and observed. For *in situ* hybridization, 3 biological repeats were performed.

The SbLOX3 cDNA fragment was inserted into a pST19 vector using the primers listed in **Supplementary Table S1**. The resulting plasmid was subjected to *in vitro* transcription to generate digoxigenin (DIG)-labeled RNA probes employing a DIG labeling kit (Roche, Switzerland). Prior to hybridization, the probes were fragmented to 100–150 bases by hydrolysis in carbonate buffer (0.04 mM NaHCO_3_ and 0.06 mM Na_2_CO_3_), then precipitated with 70% (v/v) ethanol and resuspended in diethylpyrocarbonate-treated water at a final concentration of 1 ng/μL. *In situ* hybridization was carried out according to Omori et al. (2009).

### 2.10 Activity of ROS scavenging enzyme in roots

Roots after PEG-simulated drought stress were used for activity ROS scavenging enzyme analysis. Crude enzyme solution was prepared according to the methods described by Chen et al. (2013). Activity of superoxide dismutase (SOD) was measured by the SOD inhibition of the photochemical reduction of nitro blue tetrazolium (NBT) (Dhindas et al., 1981). One unit (U) of SOD activity was defined as the amount of enzyme that caused 50% inhibition of NBT reduction under the assay conditions. Peroxidase (POD) activity was measured by the increase in absorbance at 470 nm because of the guaiacol oxidized by H2O2 (Maehly and Chance 1955). One unit (U) of POD activity was defined as the amount of enzyme required to catalyze the formation of 1 μmol tetraguaiacol per minute. MDA content was determined by the TBA reaction (Heath and Packer, 1968; Hodges et al., 1999). Three technical replicates for each of the three biological replicates were performed for each measurement.

### 2.11 Statistical analysis

All data are presented as mean ± SD. One or two-factors AVNOA analysis were used to compare group differences using SPSS 16.0. P-values < 0.05 were considered significant. For all UPLC-MS analysis, 5 biological repeats were used. For all qRT-PCR, analysis, 3 biological repeats were used. For root morphology analysis, 9 biological repeats were used.

## 3 Results

### 3.1 *SbLOX3* regulated the content of 4′-deoxyflavones in the roots of *S. baicalensis*

We evaluated the content of metabolites related to baicalin biosynthesis in transgenic *S. baicalensis* plants to determine whether *SbLOX3* is important for baicalin biosynthesis. The expression of *SbLOX3* in RNAi lines was reduced to 16%-72% of the WT level and increased to 134%-362% in overexpression lines (**Supplementary Figures S1A, B**). RNAi-6 and OE-14 lines were chosen for further research because they exhibited significantly different contents of baicalin, wogonin, and JAs compared with the WT line (**Supplementary Figure S1C**).

JAs content was analyzed in the RNAi-6 and OE-14 lines. JAs content significantly increased in the *SbLOX3* OE-14 line and decreased in the RNAi-6 line (**Figure 1A**). The contents of metabolites related to baicalin biosynthesis exhibited the same tendency (**Figure 1A**). The relative content of cinnamic acid displayed no significant differences among lines, whereas the content of other metabolites increased in the OE line and decreased in the RNAi line. The expression levels of genes related to baicalin biosynthesis also increased in the OE line and decreased in the RNAi line, except for *SbCHS-2* and *SbCHI*, which increased in the OE line but remained unchanged in the RNAi line (**Figure 1B**). These results showed that *SbLOX3* directly regulated JAs biosynthesis and indirectly regulated baicalin biosynthesis, which was extending our prior report in hairy roots (Geng et al., 2023b).

**Figure 1:**
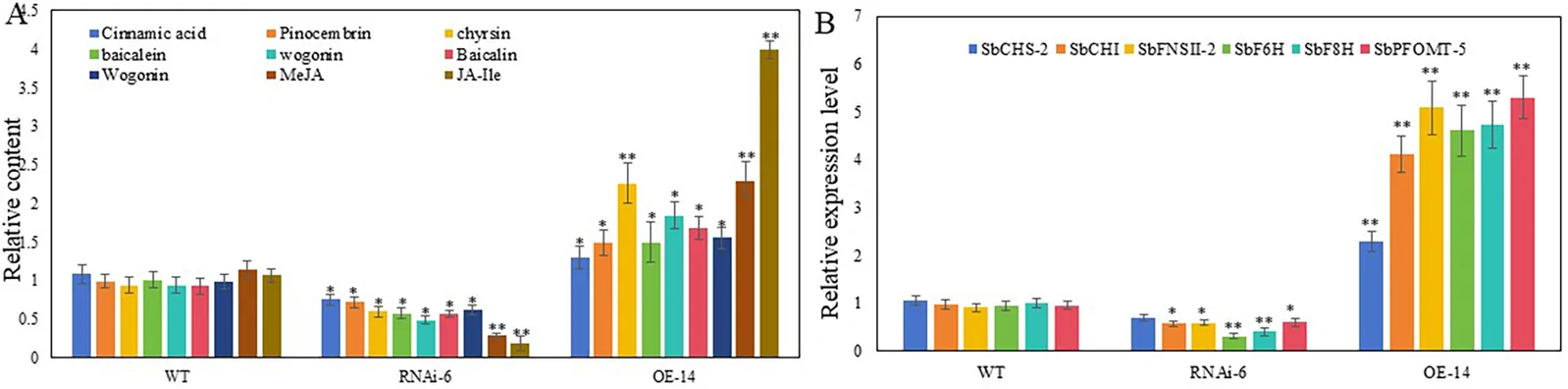
Relative content of 4′-deoxyflavones **(A)** and relative expression level of genes related to biosynthesis of 4′-hydroxyflavones **(B)** in transgenic roots of Sbaicalensis. Representation: mean ± standard deviation, * represents p<0.05, ** represents p < 0.01, ANOVA test, for metabolites content, n=5, for relative expression level, n=3.

### 3.2 SbLOX3 short-term PEG-simulated drought response in transgenic roots

Our previous study found that *SbLOX3* responded to drought stress and induced the expression of SbMYB3 and other genes related to the biosynthesis of baicalin in hairy roots. However, the expression patterns and response models might differ in whole plants. PEG-simulated drought treatment was performed on WT, RNAi, and OE lines to determine whether *SbLOX3* responds to drought stress and regulates downstream genes such as SbMYB3 in transgenic plant roots. The results showed increased expression of *SbLOX3*, which was even more pronounced in the OE-14 line. The expression of *SbLOX3* was inhibited in the RNAi-6 line (**Figure 2**). The expression of *SbMYB3* and other genes related to baicalin biosynthesis displayed the same tendency (**Figure 2**). The expression of *SbMYB3*, *SbFNSII-2*, *SbF6H*, *SbF8H*, and *SbPFOMT-5* was regulated by drought and *SbLOX3* (**Figure 2**). The expression of *SbCHS* and *SbCHI* was induced by drought stress and in the OE line, but showed no significant difference in the RNAi line (**Figure 2**).

**Figure 2:**
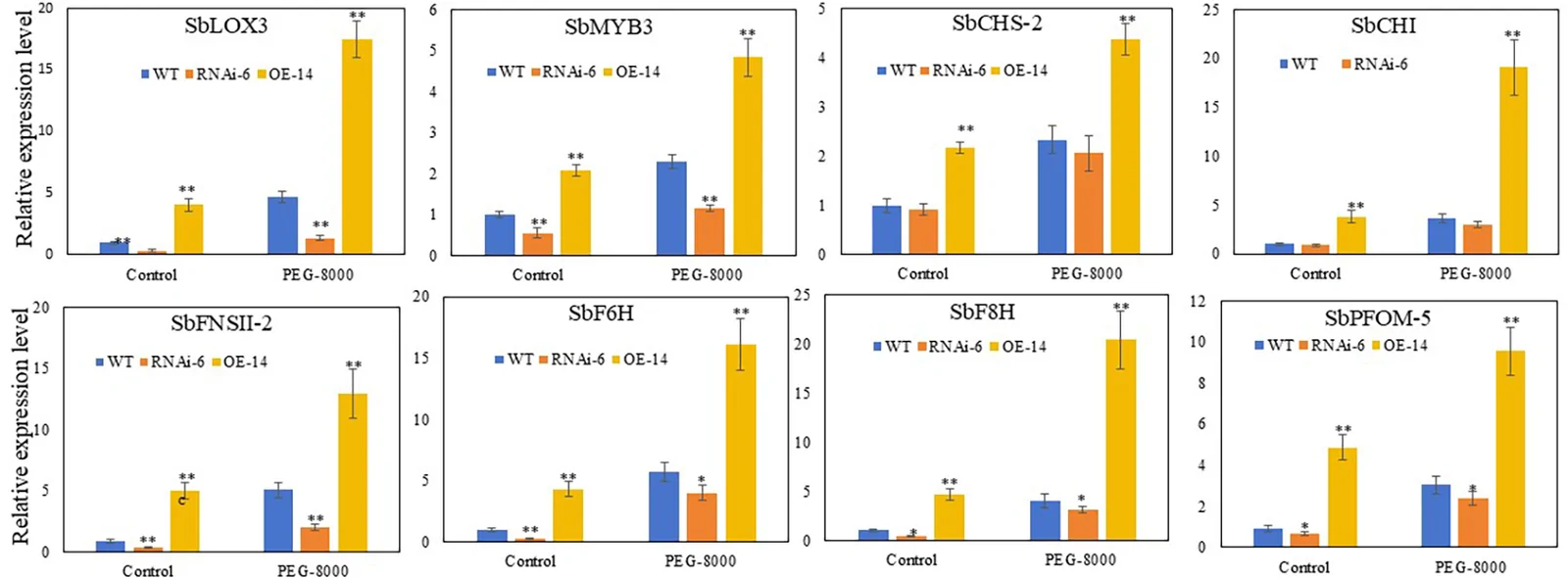
Relative expression level of genes related to biosynthesis of 4′-deoxyflavones in transgenic roots of *S.baicalens* is under PEG-simulated drought stress. Representation: mean ± standard deviation, * represents p < 0.05, ** represents p<0.01, ANOVA test, n=5.

Under control conditions, SbLOX3-OE roots showed significantly higher SOD and POD activities and lower MDA conten than WT, indicating a strengthened antioxidant capacity in the overexpression line (**Supplementary Figure S2**). PEG-8000 treatment elevated SOD and POD activities and increased MDA content in all three genotypes, but the magnitude of these changes differed markedly. Under drought, SbLOX3-OE roots retained the highest SOD and POD activities and the lowest MDA content, whereas SbLOX3-RNAi roots showed reduced SOD and POD activities and ~36% higher MDA content than WT (**Supplementary Figure S2**).

### 3.3 *SbLOX3* maintained root growth and enhanced accumulation of baicalin under Long-term drought stress

To further clarify the role of *SbLOX3* in drought resistance and baicalin biosynthesis under cultivation conditions, transgenic and nontransgenic plants were exposed to long-term water deficit (**Figure 2**). As illustrated in **Figure 3**, drought treatment markedly influenced shoot dry weight, root dry weight, and the root/shoot ratio. After two months of stress, the RNAi line showed lower shoot and root dry weights compared with the WT, leading to a reduced root/shoot ratio under drought (**Figures 3A–C**). In the OE line, the shoot and root dry weights were comparable to those of the WT, yet the root/shoot ratio was elevated under drought conditions (**Figures 3A–C**). These findings indicate that *SbLOX3* is associated with drought-responsive root phenotypes.

**Figure 3:**
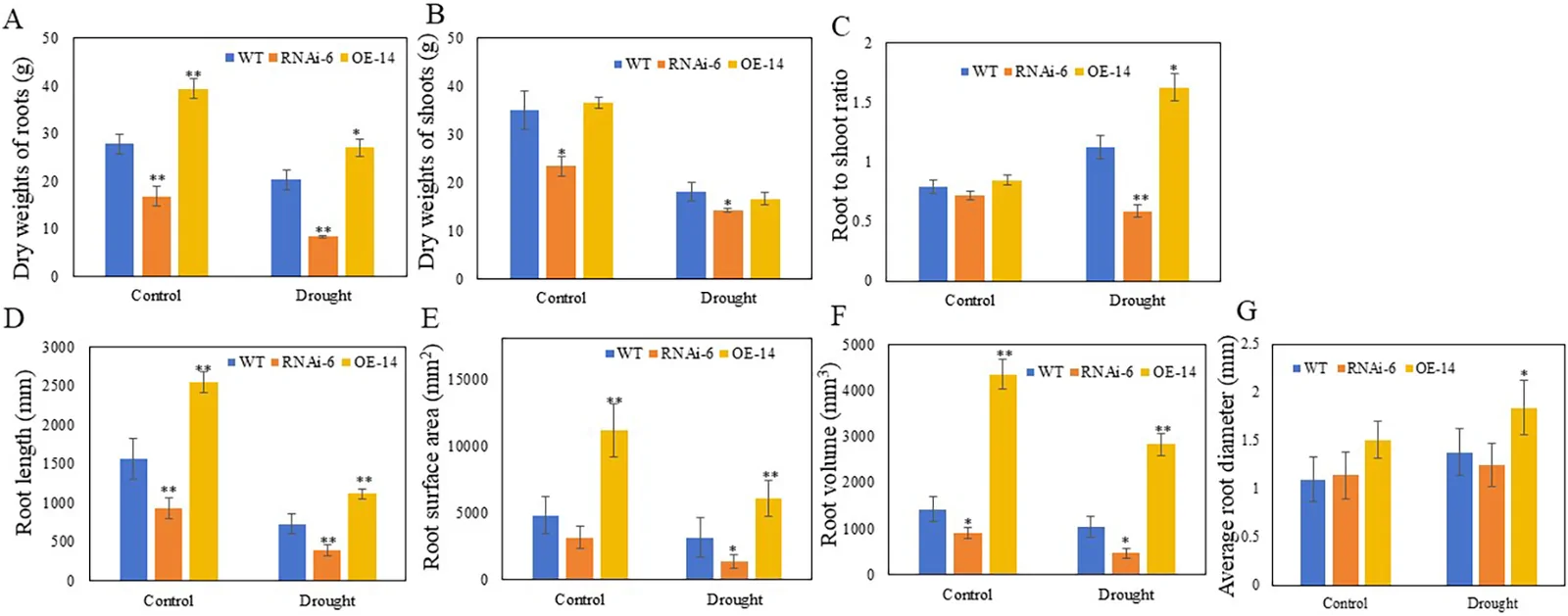
Root scan results of transgenic lines of *S.baicalens* is under long-term drought stress. **(A)** Dry weights of roots. **(B)** Dry weights of shoots. **(C)**. Root to shoot ratio. **(D)** Root lengt. **(E)** Root surface area. **(F)** Root volume. **(G)** Average root diameter Representation: mean ± standard deviation,* represents p<0.05, ** represents p<0.01, ANOVA test, n=9.

We also examined the root system to gather more insights into the changes in root morphology in response to drought stress. Measurements of total root length, total root surface area, total root volume, and average root diameter were taken to illustrate the varying responses of SbLOX3 transgenic plants.Under control conditions, the SbLOX3 RNAi line’s total root length, total surface area, and total volume were less than those in the WT line.Under control conditions, they exhibited higher levels in the OE line compared to the WT line.The phenotypic differences among the RNAi, OE, and WT lines were even more pronounced in the presence of drought stress (**Figures 3D–F**). Under control and drought conditions, the average root diameter did not differ significantly between lines (**Figure 3G**).

To determine whether SbLOX3 overexpression confers drought tolerance beyond its constitutive growth effect, we performed two-way ANOVA with genotype, treatment, and genotype × treatment interaction as sources of variation for all six phenptype in **Figure 3** (**Table 1**). All six traits showed showed significant genotype × treatment interactions (p < 0.01), indicating that the genotypes responded to drought stress.

**Table 1:** Two-way AVONA of genotype, treatment, and genotype × treatment of six phenotype related to drought stress.

Phenotype	Genotype_F	Genotype_p	Treatment_F	Treatment_p	Interaction_F	Interaction_p
Dry weight of shoots (g)	246.0365175	5.90E-26	911.4097088	7.10E-33	11.93178665	6.22E-05
Dry weight of root (g)	398.3710236	1.28E-30	763.7460946	3.94E-31	114.8209904	5.08E-19
Root to shoot ratio	59.36822852	1.05E-13	3.74931411	0.058729081	53.07590511	6.90E-13
Root length (mm)	161.0974501	5.10E-22	336.4571783	2.51E-23	23.62531839	7.19E-08
Root surface area (mm^2^)	106.9607255	2.06E-18	78.48272137	1.14E-11	7.359886179	0.001629605
Root volmue (mm^3^)	847.58562	3.61E-38	165.3056094	3.68E-17	36.05330555	2.76E-10

Water deficit enhanced baicalin accumulation in *S. baicalensis* roots. To verify the involvement of *SbLOX3* in this drought-induced accumulation, the relative levels of metabolites from the baicalin biosynthetic pathway were examined. Cinnamic acid content did not differ significantly among the lines, yet it was strongly upregulated by drought. Pinocembrin, chrysin, baicalein, wogonin, and baicalin all showed elevated levels in the OE line and under water deficit; in the RNAi line, these metabolites were reduced under drought and remained unchanged under control conditions (**Figure 4**). JAs content was significantly higher under drought stress and in the OE line, and was lower in the RNAi line under both conditions (**Figure 4**).

**Figure 4:**
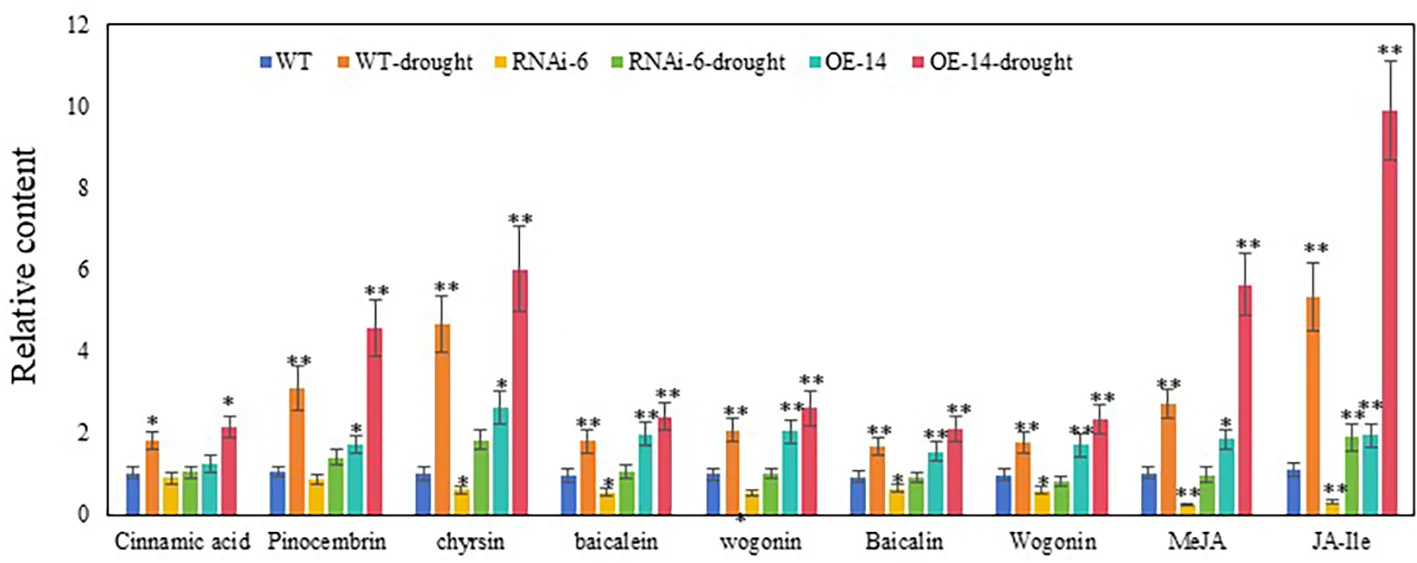
Relative content of metabolites related to 4′-deoxyflavones biosynthesis in transgenic roots of *S.baicalens* is under long-term drought stress. Representation: mean ± standard deviation, * represents p<0.05, ** represents p<0.01, ANOVA test, n=5.

### 3.4 *SbLOX3* expression was induced in the xylem and cambium in *S. baicalensis*

WT roots were subjected to *in situ* hybridization under control and long-term drought treatments to confirm the tissue-specific expression of *SbLOX3* under drought stress. The results showed strong expression of *SbLOX3* under drought stress in xylem, cambium, and phloem, SbLOX3 was expressed primarily in the xylem with weak signals in the cambium under control conditions (**Figures 5A, B**). No hybridization signal was observed in sense-probe controls (**Figures 5C, D**), confirming the specificity of the antisense-probe signal. These results indicated that drought stress induced the expression of *SbLOX3* in xylem, cambium, and phloem, promoted JAs biosynthesis under drought stress, and enhanced the accumulation of baicalin.

**Figure 5:**
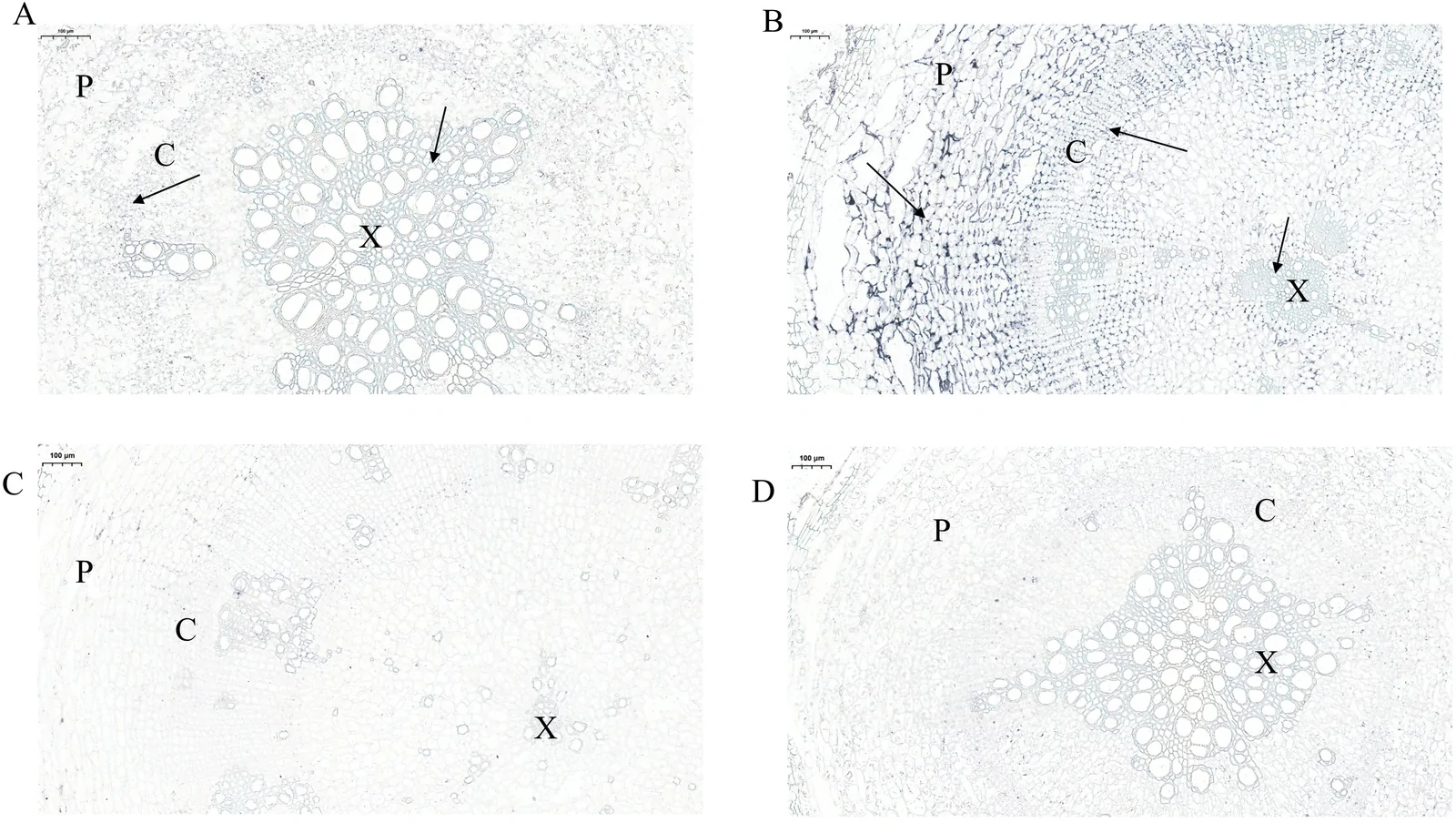
*In situ* hybridization of SbLOX3 under long-term drought stress in WT roots. **(A)** Roots under control conidiation with anti-sense probe. **(B)** Root under long-term drought stress conidiation with anti-sense probe. **(C)** Roots under control conidiation with sense probe. **(D)** Root under long-term drought stress conidiation with sense probe. X, xylem. C, cambium. (P) phloem. Bar: 100μm. Arrow reparent positive signals.

### 3.5 *SbLOX3* overexpression and drought stress led to an earlier root hollow development in *S. baicalensis*

WT, RNAi-6, and OE-14 lines (20 months old) were subjected to drought stress to confirm the contribution of *SbLOX3* and JAs to root hollow development. (**Supplementary Figure S3**) After 3-month moderate drought treatment, the roots were harvested and longitudinal sections through the center were prepared to observe root hollow development (**Figure 6**). The results showed that 7 of 18 seedings under drought stress in *SbLOX3* overexpression lines were observed with root hollow development.(**Figure 6**). No root hollow development was observed in WT and RNAi lines in the control line or under drought stress (**Figure 6**). These observations are consistent with the interpretation that the combination of SbLOX3 overexpression and long-term water deficit is associated with precocious root hollow formation.

**Figure 6:**
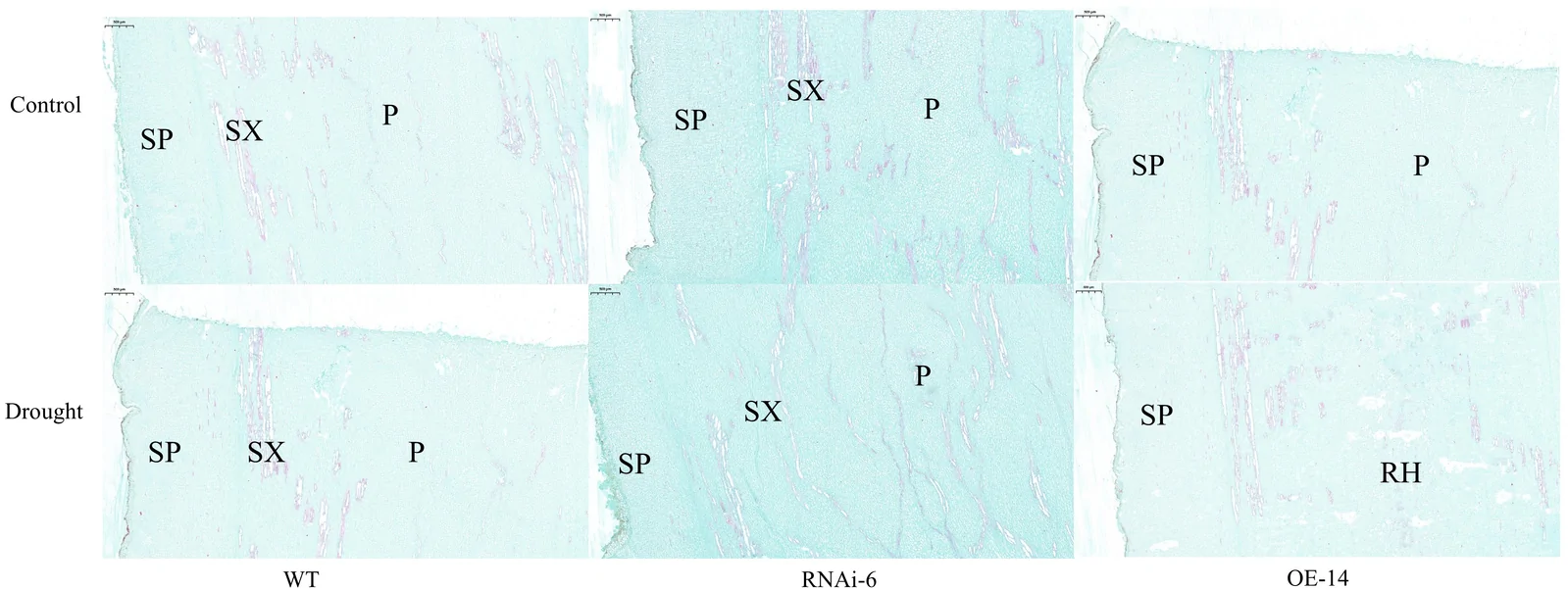
Anatomical structure of 20-month-old transgenic roots of *S.baicalensis* after long-term drought stress. RH, root hollow. P, pith. SX, secondary xylem. SP, secondary phloem. Bar: 500μm.

## 4 Discussion

### 4.1 *SbLOX3* regulated the biosynthesis of baicalin by modulating JAs biosynthesis

Earlier work suggestedf that SbLOX3 controls JAs production and indirectly modulates baicalin biosynthesis in *S. baicalensis* hairy roots (Geng et al., 2023b). In the present study, we established SbLOX3-overexpressing and RNAi transgenic lines (**Supplementary Figure S1**) and verified that, in the roots of intact transgenic plants, SbLOX3 directs JAs synthesis and influences the formation of baicalin (**Figure 1**).

In the roots of S. baicalensis, JAs levels controlled baicalin production. JAs treatment induced the expression of *SbWRKY75* and *SbWRKY41*. SbWRKY75 directly targeted the flavonoid biosynthetic gene *SbCLL-7*, whereas SbWRKY41 directly targeted two other genes, *SbF6H* and *SbUGT*, thereby modulating baicalin biosynthesis (Fang et al., 2023). Earlier, Hu et al. (2022) and Yuan et al. (2013) showed that MeJA application also upregulated genes associated with baicalin biosynthesis.

We observed that *SbCLL-7* and *SbF6H* transcript levels were upregulated in the *SbLOX3*-OE line and downregulated in the *SbLOX3*-RNAi line, while JAs content was correspondingly higher in the overexpressor and lower in the RNAi line (**Figure 1**). These results align well with previously published data.

### 4.2 *SbLOX3* responded to drought stress and enhanced drought resistance in the roots of *S. baicalensis*

Under drought stress, both the transcript level of *SbLOX3* and JAs content were elevated, accompanied by the upregulation of baicalin biosynthesis-related genes, including *SbFNSII-2*, *SbCHS-2*, *SbCHI*, *SbF6H*, *SbF8H*, and *SbPFOMT-5* (**Figure 2**). The increased expression of *SbLOX3* promoted drought resistance in *S. baicalensis* roots. Compared with the WT, the *SbLOX3*-OE line exhibited greater root length, root dry weight, root surface area, root volume, and root-to-shoot ratio under prolonged water deficit, whereas these parameters were diminished in the *SbLOX3*-RNAi line (**Figure 3**). Collectively, these data suggest that *SbLOX3* positively modulates root drought tolerance in *S. baicalensis*.

Jasmonates (JAs) act as essential signaling molecules that coordinate plant drought responses and developmental processes (Eyidogan et al., 2012). Under water deficit, JAs reinforces the antioxidant defense system, thereby attenuating reactive oxygen species (ROS)-mediated oxidative damage (Shikha et al., 2023). JAs also favors root growth while suppressing shoot growth to conserve water (Sonatan et al., 2026), resulting in elevated root dry weight and a higher root-to-shoot ratio. In *Scutellaria baicalensis* roots, JAs are known to regulate 4′-deoxyflavone biosynthesis (Geng et al., 2023a; Wang et al., 2022), and the accumulation of these metabolites is likewise modulated by abiotic stress (Yeo et al., 2022; Su et al., 2018).

Our data suggest that SbLOX3 modulates JAs accumulation in S. baicalensis roots (**Figure 1**). Overexpression of SbLOX3 elevated JAs production, which conferred drought tolerance and stimulated root growth under water deficit (**Figure 3**). In contrast, RNAi-mediated suppression of SbLOX3 reduced JAs levels, resulting in increased drought susceptibility of the roots (**Figure 3**).

### 4.3 Root hollow development was induced by drought stress and *SbLOX3*, resulting in the accumulation of 4′-deoxyflavones

The content of 4′-deoxyflavones was upregulated under long-term drought stress compared with control conditions (**Figure 4**). The content was higher in the *SbLOX3* OE line than in the WT line and lower in the *SbLOX3* RNAi line than in the WT line (**Figure 4**). Metabolites related to the biosynthesis of 4′-deoxyflavones exhibited the same tendency (**Figure 4**). The biosynthesis of 4′-deoxyflavones was induced under drought stress in the roots of *S. baicalensis*. The expression of *SbbHLH74*, *SbbHLH98*, and *SbbHLH142* was regulated by *SbbHLH5* and induced 4′-deoxyflavone biosynthesis under drought stress (Sun et al., 2025). Li et al. (2025) discovered that *SbBZR1* expression was induced by drought stress and further induced the expression of *SbPAL*, *SbCHS*, and *SbCHI*, increasing the content of 4′-deoxyflavones. Chen et al. (2025) discovered a drought-response MYB transporter *SbMYB8*, which induced the biosynthesis of 4′-deoxyflavones. We verified the validity of these conclusions through long-term drought treatment in the field environment.

*SbLOX3* expression was also induced by drought stress (**Figure 1**), and the upregulation of *SbLOX3* expression could still be maintained after 2 months of long-term drought stress (**Figure 5**). *SbLOX3* was expressed primarily in the xylem with weak signals in the cambium under control conditions, whereas *SbLOX3* was strongly expressed in the xylem, cambium, and phloem under drought stress (**Figure 5**). These results indicated that *SbLOX3* was related to 4′-deoxyflavone biosynthesis under drought stress. This is because 4′-deoxyflavones are stored in the phloem.

*SbLOX3* expression was induced by drought (**Figure 1**) and remained elevated after 2 months of sustained water deficit (**Figure 5**). *In situ* hybridization localized SbLOX3 transcripts primarily in the xylem with weak signals in the cambium under control conditions, and additionally to cambium and phloem under drought (**Figure 5**). These results are consistent with SbLOX3 was related to 4′-deoxyflavone biosynthesis′-deoxyflavone biosynthesis under drought.

Root hollows developed in the SbLOX3-OE line at 20 months of age under drought, but not in WT or RNAi lines under either treatment (**Figure 6**). Under standard cultivation, hollowing normally begins in the spring following the second year of growth (Geng et al., 2023a); therefore, the OE × drought combination represents a precocious phenotype rather than a typical developmental event. This observation coincided with a marked elevation of JA-Ile—the only established jasmonate species capable of activating the COI1-JAZ co-receptor (Fonseca et al., 2009; Sheard et al., 2010)—in SbLOX3-OE roots and a corresponding reduction in SbLOX3-RNAi roots relative to WT (**Figure 1A**). This provides a more direct biochemical link between SbLOX3 activity and jasmonate signaling than the previously reported JA or MeJA measurements (Geng et al., 2023a, 2023b). Nevertheless, whether and how elevated JA-Ile contributes mechanistically to hollow formation remains to be determined.

## 5 Conclusions

In conclusion, drought stress in *S. baicalensis* roots activates *SbLOX3* expression, enhancing JAs biosynthesis. The elevated JAs signaling upregulates the expression of key transcription factors and structural genes in the flavonoid pathway, such as *SbFNSII-2*, *SbF6H*, and *SbF8H*, thereby promoting the accumulation of defensive flavonoids, primarily 4′-deoxyflavones. Concurrently, the sustained activation of *SbLOX3*, particularly in the xylem and cambium under long-term drought stress, cooperates with JAs to induce the precocious development of root hollows. This drought-associated anatomical alteration are associated with enhanced drought tolerance and increased accumulation of measured flavonoids.

## Data Availability

The original contributions presented in the study are included in the article/**Supplementary Material**. Further inquiries can be directed to the corresponding authors.
